# Can chatGPT-4o reliably standardize PSMA PET/CT and PET/MRI reports using PROMISE V2 criteria? - An exploratory study

**DOI:** 10.1186/s13550-026-01475-z

**Published:** 2026-06-26

**Authors:** Anna Hinterberger, Maurin H. Mangold, Caroline Weigel, Henri Hartmann, Dominik Nörenberg, Matthias F. Froelich, Ricarda Ebner, Caelán Max Haney-Aubert, Karl-Friedrich Kowalewski, Stefan O. Schönberg, Freba Grawe

**Affiliations:** 1 DKFZ Hector Cancer Institute at the University Medical Center Mannheim, Heidelberg, Germany; 2 Junior Clinical Cooperation Unit Translational Molecular Imaging in Oncologic Therapy Monitoring (E310), German Cancer Research Center, Heidelberg, Germany; 3 Junior Clinical Cooperation Unit Intelligent Systems and Robotics in Urology (ISRU), German Cancer Research Center, Heidelberg, Germany; 4 Department of Urology and Urologic Surgery, University Medical Centre Mannheim, University of Heidelberg, Mannheim, Germany; 5 Department of Radiology and Nuclear Medicine, University Medical Center Mannheim, Heidelberg University, Mannheim, Germany; 6 Department of Radiology, LMU University Hospital, LMU Munich, Munich, Germany; 7 German Cancer Research Center (DKFZ), Im Neuenheimer Feld 280, 69120 Heidelberg, Germany

**Keywords:** PROMISE, ChatGPT4o, miTNM, Prostate cancer

## Abstract

**Background:**

Structured reporting standardizes and facilitates reporting, improves accurate communication, and ultimately clinical decision-making. Although standardized frameworks such as PROMISE criteria are available for prostate-specific membrane antigen positron emission tomography (PSMA PET) for prostate cancer patients, free-text reporting remains predominant in both clinical routine and trials. Large language models (LLMs) may enable low-effort, time-efficient extraction of structured classifications from narrative reports. This study evaluated the performance of ChatGPT-4o for extracting PROMISE V2-based classifications from unstructured PSMA-PET/CT and PET/MRI reports.

**Results:**

For PSMA-PET/CT, overall miTNM accuracy was 79.8%, whereas PSMA-PET/MRI achieved a significantly higher accuracy of 91.0% (OR = 2.80, 95% CI: 1.32–6.51, *p* = 0.003). Component-wise, PET/MRI outperformed PET/CT in T-stage classification (83.8% vs. 57.7%; OR = 3.83, 95% CI: 1.34–12.69, *p* = 0.006) and demonstrated numerically higher N-stage classification accuracy (100% vs. 85.9%, *p* = 0.014), while M-stage classification was comparable between modalities (89.1% vs. 95.7%; OR = 0.84, 95% CI: 0.20–4.19, *p* = 0.748). PRIMARY score accuracy was also comparable for PET/CT and PET/MRI (70.4% vs. 88.1%; OR = 0.43, 95% CI: 0.05–2.14, *p* = 0.315). ChatGPT-4o’s rationale for classifications was rated highly plausible across modalities, with a minimum Likert score of ≥ 4.8 for miTNM and 4.1 for PRIMARY.

**Conclusion:**

ChatGPT-4o enables reliable extraction of PROMISE V2–based N- and M-stage classifications from free-text PSMA-PET reports, with limited accuracy for T-stage. This work provides a first step toward leveraging LLMs to support structured and efficient reporting in PSMA PET imaging and points out present limitations.

**Supplementary Information:**

The online version contains supplementary material available at 10.1186/s13550-026-01475-z.

## Introduction

 Large language models (LLMs) are advanced artificial intelligence systems trained on large-scale text datasets to process and generate human language. Recent LLM-based models, such as the Generative Pre-Trained Transformer (GPT) developed by OpenAI, have demonstrated expert-level performance in a wide range of language-based tasks across different domains [1, 2]. These capabilities have sparked increasing interest in radiology, particularly in the context of structured reporting, which is known to guide image interpretation, enhance reproducibility, and facilitate communication with referring clinicians [3–5]. However, the integration of LLMs into imaging workflows remains limited.

For the disease extent of prostate cancer, the PROMISE (Prostate Cancer Molecular Imaging Standardized Evaluation) framework, which was updated in 2023 (V2), was developed to standardize the interpretation of prostate-specific membrane antigen (PSMA) targeted positron emission tomography (PET) [6]. PROMISE provides a unified system for comparing imaging, biopsy, and biomarker findings. It forms the basis for the molecular imaging Tumour-Node-Metastasis (miTNM) classification, which is a molecular imaging-based analogue to the conventional TNM system for tumour staging and includes the PRIMARY score, which characterizes intraprostatic PSMA uptake patterns to guide local staging and biopsy targeting [7]. Since its introduction, PROMISE has been validated in multiple studies and is associated with improved diagnostic accuracy and consistency in staging and treatment planning for prostate cancer [8–10]. Moreover, the PROMISE criteria demonstrated their potential to enable precise risk stratification for overall survival prediction in both early and advanced stages of prostate cancer [11]. Despite these advantages, structured reporting frameworks such as PROMISE have not been implemented in routine clinical practice nor systematically in clinical trials. Free-text reporting remains predominant, and the transition to structured formats often requires training, time, and system-level changes [12, 13]. To overcome these challenges, LLMs may offer a scalable, low-effort solution by enabling real-time extraction of standardized classifications from narrative reports or by assisting in automated structured reporting [5, 14]. However, before clinical integration, such tools must be thoroughly validated for accuracy, reliability, and reproducibility in a medical context [15, 16].

In this study, we evaluated the feasibility of ChatGPT-4o, accessed via the ChatGPT interface, to extract PROMISE V2-based miTNM classifications and PRIMARY scores from unstructured PSMA-ligand PET/CT and PET/MRI reports. Given that MRI offers superior soft-tissue contrast for local staging of prostate cancer compared to CT, we further hypothesized that ChatGPT-4o would show improved performance when interpreting PET/MRI reports.

## Methods

### Study design and patient cohort

In this single-center retrospective study, 137 free-text PSMA-PET reports (written in German) from 137 consecutive patients with histologically confirmed prostate cancer were screened. Ten reports were used for prompt development and were excluded from further analysis. An additional 19 patients were excluded due to missing clinical information, resulting in a final study cohort of 108 patients. The cohort included 71 PSMA-PET/CT and 37 PSMA-PET/MRI scans performed between January 2021 and December 2024. Indications comprised initial staging, biochemical recurrence, and therapy monitoring. Ethics approval was obtained from the local institutional review board (EK II 2023-886-AF 11).

### Imaging protocols

PET/CT and PET/MRI scans were acquired using standard institutional protocols.

### PSMA PET/CT imaging

PET imaging was performed according to established institutional protocols according to current guidelines using [¹⁸F]PSMA. Low-dose CT for attenuation correction was acquired with 10 mA, 120 kV, 512 × 512 matrix. After intravenous injection of contrast agent (*n* = 33) diagnostic CT scans of the neck, thorax, abdomen, and pelvis (100–190 mAs; 120 kV) were acquired. Patients received diagnostic CT scans without contrast enhancement (*n* = 38) in case of known allergic reactions to iodinated contrast agent, renal impairment/failure, or hyperthyroidism or because contrast enhanced CT was already performed up to two weeks prior PET/CT. All acquired PET/CT scans were analysed using a dedicated software package (syngo.via, Siemens Healthcare).

### PSMA PET/MRI imaging

18 patients received [^18^F]PSMA and 19 patients received [^68^Ga]PSMA according to established institutional protocols. In parallel, MRI included T2-HASTE, DWI, and contrast-enhanced T1-VIBE sequences, which were acquired in all patients. PET data were acquired in 3D mode. The raw data was reconstructed iteratively and evaluated using the ROI technique (regions of interest) and determination of the SUV (Standardized Uptake Value).

### PROMISE extraction via ChatGPT-4o prompting

All reports were manually extracted from the radiology information system (RIS) by medical staff including radiotracer type and dose, the body of the report text and the conclusion of the report without patient related information (e.g., name, birth date). All PSMA-PET/CT and PET/MRI reports were additionally reviewed by one investigator to ensure completeness for quality proposes. Findings in the report were minimally adjusted to anonymize the report. A standardized prompt was iteratively developed based on ten representative raw text reports (which were not further included in the dataset) and optimized for accurate extraction of PROMISE V2-based classifications. The prompt was evaluated in both German and English to assess whether language-specific semantic interpretations or subtle, staging-relevant nuances in the German language influenced the output. Moreover, we performed repeated test runs on identical reports to investigate the stability of the web-application. No differences in the output were observed between the two languages and the multiple test-runs on identical reports, leading to the decision to continue with the web-application rather than switching to the Application Programming Interface (API). The web-application is an accessible, state-of-the-art commercial model and could be readily implemented in clinical practice without specialized hardware or technical infrastructure, offering a suitable option for our exploratory study. Every report was submitted to ChatGPT-4o as a raw text in addition to the prompt. Moreover, all reports were submitted to ChatGPT-4o in one day to ensure that changes on the version due to ChatGPT updates do not influence the output. To reflect routine clinical workflow, we deliberately chose a simplified staging approach. Therefore, both the expert reference reading and the ChatGPT-based extraction followed an AJCC-oriented hierarchical interpretation.

### Prompt for miTNM classification

Given the following PSMA-PET report, classify findings using PROMISE V2 published by Robert Seifert et al. in 2023. Provide the PROMISE classification (only include the following categories for local tumour: miT0, miT2 (u and m) and miT3 (a and b), miT4, miTr; for intrapelvic nodes: miN0, miN1, miN2; for distant metastases: miM0, miM1 (a, b and c) a short rationale based on the report text why you chose this PROMISE classification. Only state the result and the rational. Only state the highest scores for each category T, N and M. Please report results in English.

### Prompt for PRIMARY classification

“Given the following PSMA-PET report, classify findings using the table attached and a short rationale why you chose this PRIMARY score. Only state the result and the rational. Please report results in English.”

PRIMARY score.

1: No dominant intraprostatic pattern on PSMA. Low grade activity, miT0.

2: Diffuse transition zone activity or symmetrical central zone activity that does not extend to the prostate margin on CT background, miT0.

3: Focal transition zone activity visually twice above background, miT2, miT3, or T4.

4: Focal peripheral zone activity (no minimum intensity), miT2, miT3, or T4.

5: Intense uptake (visual very high intensity or SUVmax > 12), miT2, miT3, or miT4.

For PRIMARY scoring, only patients without prior prostatectomy were included to ensure intraprostatic lesion assessment. Each report was then entered into the ChatGPT-4o interface (Open AI, San Francisco, CA, USA) together with the final version of the prompt via the official web application. ChatGPT-4o generated a structured output including the miTNM classification, PRIMARY score (if applicable), along with a free-text rationale for each decision. No patient-identifying information or external datasets were uploaded. All outputs were exported into Excel (Microsoft, version 16.96.1) for further analysis.

### Reference standard and validation

**Fig. 1:**
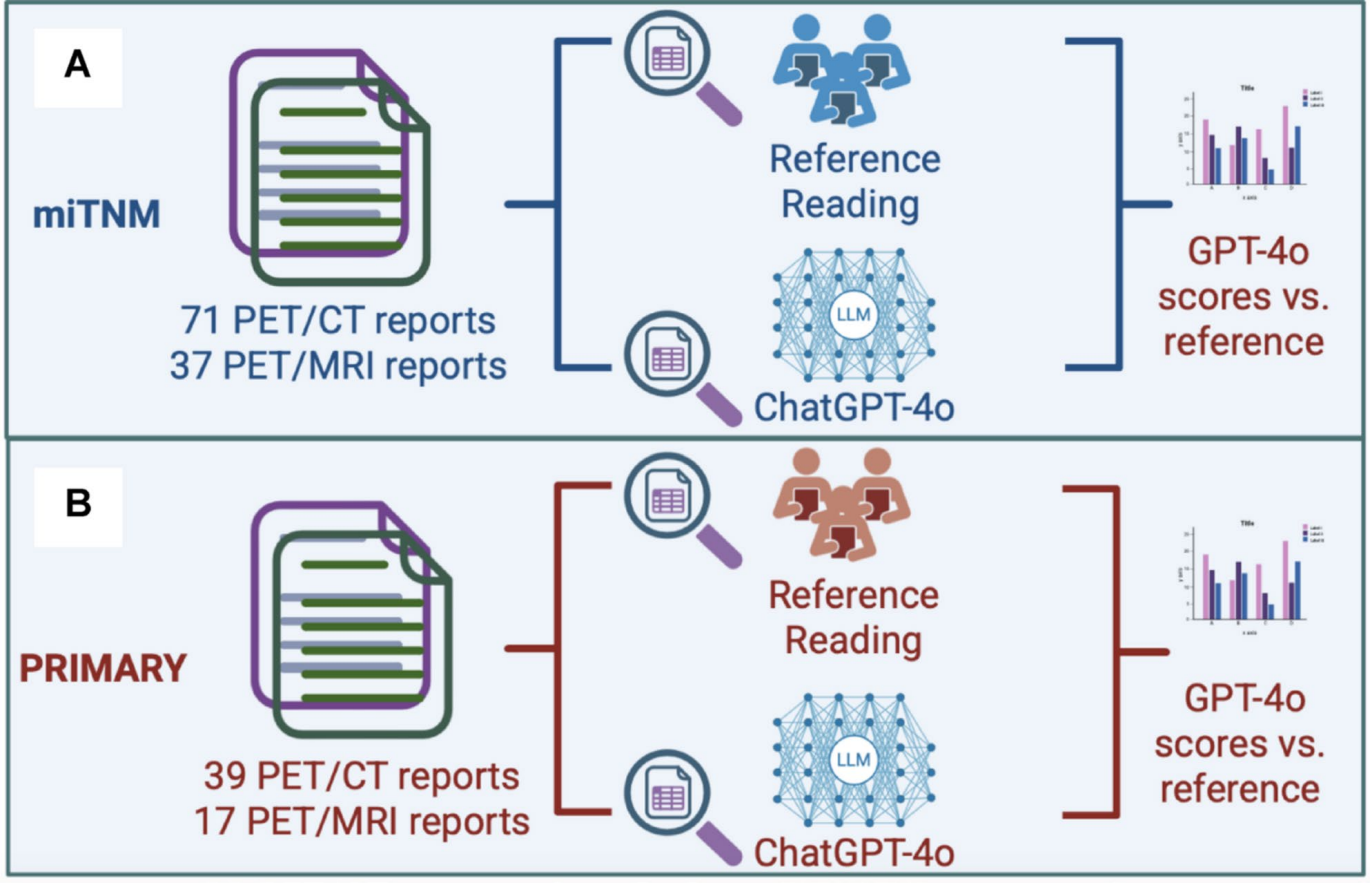
Workflow-diagram. 108 PSMA-PET (71 PET/CTs and 37 PET/MRIs) written reports were analysed. For miTNM classification, all 108 reports were included (**A**). For PRIMARY classification, 56 reports of patients without prior prostatectomy were included (**B**). PRIMARY and miTNM was assessed based on the output of ChatGPT-4o and human reading, using the information provided in the free-text-reports

For comparison of results between ChatGPT-4o, all written reports were classified according to PROMISE V2 for PRIMARY and miTNM by one radiologist with expertise in hybrid imaging and one urologist specialized (= human reading) in prostate cancer, with additional validation by a board-certified nuclear medicine physician. Figure 1 depicts the extraction and validation process. For further analysis, instances of underestimation and overestimation were evaluated. Underestimation was defined as ChatGPT-4o assigning a lower T, N, or M category than the human reader, whereas overestimation was defined as ChatGPT-4o assigning a higher T, N, or M category than the human reader.

### Evaluation of model output

In addition to classification accuracy, the ChatGPT-4o generated rationales for each miTNM component and PRIMARY scoring, which was independently evaluated by two readers using a 5-point Likert scale (1 = poor, 5 = excellent) to assess plausibility and factual correctness.

### Statistical analysis

For accuracy of ChatGPT-4os miTNM and PRIMARY classification the percentual correctness, based on the human reading, and 95% confidence interval (CI) was calculated. Accuracy was compared between imaging modalities (PET/CT vs. PET/MRI) and clinical indication (initial staging vs. recurrence) using the Fisher’s exact test. Likert ratings of ChatGPT-4o’s rationales were obtained independently by two readers and averaged, results were calculated based on a two-tailed t-test. Data are presented as mean and standard deviation (SD). Cohens kappa was calculated for ChatGPT-4o and the validation set as well as between the two readers (M.M. and A.H.). Agreement was considered poor for κ < 0.4, fair for κ between 0.4 and 0.59, good for κ between 0.6 and 0.74, and excellent for κ > 0.74. Results were considered significant if *p* < 0.05. Statistical analyses were performed using RStudio (PositPBC, Boston, Massachusetts, US, Version 2024.12.1).

## Results

### Baseline patient characteristics

In total, 108 reports were included (71 PET/CT, 37 PET/MRI).

**Table 1:** Summarizes patient characteristics

Patient characteristics	
mean age in years ± SD	70 ±7.7
mean PSA in ng/ml ± SD	11.1 ± 30.7
Gleason score (n, %)	
Gleason 6	6 (5.5)
Gleason 7	55 (50.9)
Gleason 8	17 (15.7)
Gleason 9	26 (24.2)
Gleason 10	4 (3.7)
**Clinical/pathological TNM**	
R0	10 (16.0)
T1 cT1 pT1	25 (22.6) 20 5
T2 cT2 pT2	38 (34.4) 21 17
T3 cT3 pT3	31 (23.4) 12 19
T4 cT4 pT4	4 (3.6) 2 2
N0 cN0 pN0	72 (69.3) 42 30
N1 cN1 pN1	32 (30.7) 20 12
M0 cM0 pM0	75 (69.5) 42 33
M1a cM1a pM1a	15 (13.8) 9 6
M1b cM1b pM1b	16 (14.9) 9 7
M1c cM1c pM1c	2 (1.8) 2 0
**Clinical indications for PET imaging**	
initial staging	56 (51.8)
biochemical recurrence	52 (48.2)
after prostatectomy	39(75.0)
after radiation therapy	7 (13.5)
after combination of both or other	6 (11.5)

Characteristics of patients undergoing PSMA-PET/CT and PSMA-PET/MRI between 2021 and 2024. SD: standard deviation; PSA: prostate specific antigen; T: tumour; N: nodes; M: metastases; R0: no residual tumour after therapy. Gleason scores were derived only from histopathology of TRUS-guided biopsy specimens

### PROMISE classification extracted from written PET/CT reports

In 71 PET/CT reports, overall accuracy of miTNM classification by ChatGPT-4o from written reports compared to human readers was 79.8% (95%CI: 0.74–0.85). By component, percentage of correctly classified miTNM was 57.7% for miT (95%CI: 0.45–0.68), 85.9% for miN (95%CI: 0.77–0.92), and 95.7% for miM (95%CI: 0.90-1.00).

**Fig. 2:**
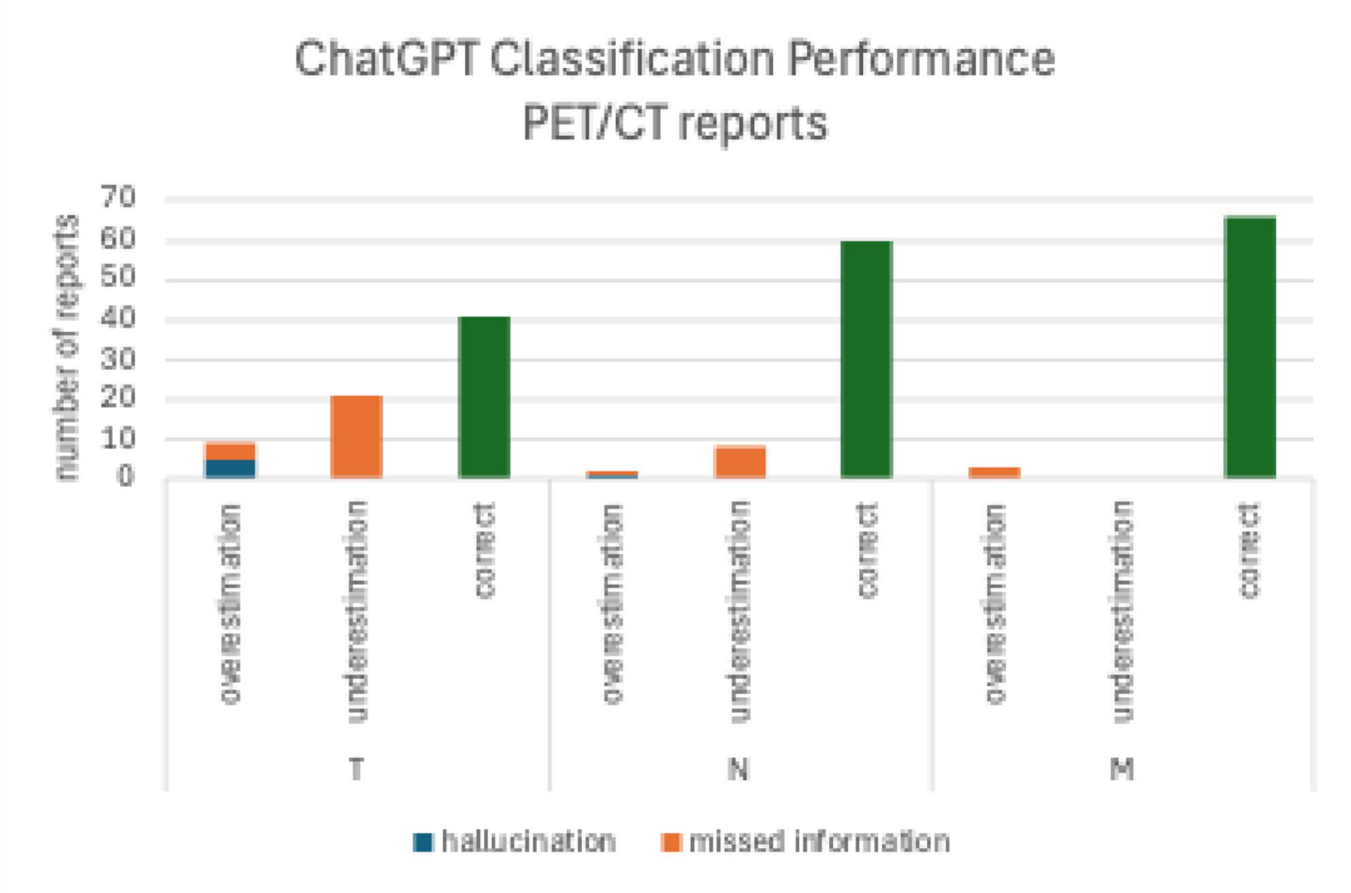
Performance of ChatGPT-4o for molecular imaging (mi) tumour (T), node (N) and metastases (M) classification applied on PSMA PET/CT reports

ChatGPT-4o *underestimated* 21 miT, 8 miN, and 3 miM categories and *overestimated* 9 miT, 2 miN. Most T-stage underestimations resulted from information that was not explicitly stated in the reports, nevertheless, these misclassifications were mostly minor shifts as T2m was often rated as T2u by ChatGPT-4o (see Table 2). We found that misclassification, particularly of miT2, was predominantly driven by information-limited discordances rather than true model-related errors. For further details, see Supplementary Table S1. N- and M-stage misclassifications similarly arose from subtle or insufficiently detailed descriptions that ChatGPT-4o did not capture (see Fig. 2). For miT, in 17 cases the most common source of error (*n* = 23) was that multifocal (m) or unifocal (u) tracer uptake was not explicitly described but only implied though phrases such as “tracer uptakes in projection of the prostate” or “tracer uptake in projection on the prostate.” In five cases, ChatGPT-4o produced hallucinated information, and in two cases therapy-related details were not recognized because the report only stated “R1”, without explicitly mentioning prostatectomy. For miN, eight cases were underestimated because ChatGPT-4o did not capture subtle wording that implied multiple lymph node metastases (e.g. “all the PET-positive lymph nodes in the small pelvis”) or vague formulations such as “possible lymph node metastases”. As a result, one N category was underestimated due to insufficiently recognized information and another due to hallucination. For miM, three cases were underestimated because ChatGPT-4o overlooked ambiguous descriptions such as “possible ganglion or lymph node adjacent to the abdominal aorta” or “multiple paraaortic lymph nodes”. In both examples, the expert reader rated the findings as miM1a as the possible lymph node adjacent to the aorta showed a SUVmax of 6.0 and should be seen as suspicious. Also the multiple paraaortic lymph nodes were described as enlarged with high SUVmax, leading to the decision of rating them as miM1a by the expert reader. For miT, the interreader agreement between the two readers from PET/CT reports was κ = 0.42 (CI: 0.25–0.58). The agreement was κ = 0.80 (CI: 0.64–0.93) for miN and κ = 0.79 (CI: 0.65–0.92) for miM. Agreement between ChatGPT-4o and the validation reading was κ = 0.38 (CI: 0.23–0.53) for miT, κ = 0.69 for miN (CI: 0.55–0.84), and κ = 0.90 (CI: 0.79–1.0) for miM.

**Table 2:** ChatGPT-4o vs. human validation reading for molecular imaging (mi) tumour (T) classification applied on PSMA PET/CT reports

PSMA PET/CT			Incorrect predictions									
Validation	Total count	**Correct prediction**	Overestimations					Underestimations				
			T2u	T2m	T3a	T3b	T4	T0	T2u	T2m	T3a	T3b
T0	5	**5**	0	0	0	0	0	0	0	0	0	0
Tr	3	**1**	**2**	0	0	0	0	0	0	0	0	0
T2u	17	**15**	0	**2**	0	0	0	0	0	0	0	0
T2m	40	**14**	0	0	**2**	**3**	0	0	**21**	0	0	0
T3a	0	na	na	na	na	na	na	na	na	na	na	na
T3b	3	**3**	0	0	0	0	0	0	0	0	0	0
T4	3	**3**	0	0	0	0	0	0	0	0	0	0

In the 39 PET/CT reports eligible for PRIMARY scoring (local tumor present), ChatGPT-4o achieved correct ratings in 70.4% (95%CI: 0.50–0.85) of patients with an interreader agreement of κ = 0.77 (CI: 0.16–0.95) when comparing the validation reading with ChatGPT-4o.

### PROMISE classification extracted from PET/MRI reports

In 37 PET/MRI reports, overall miTNM classification accuracy was at 91.0% (95%CI: 0.84–0.94). Component-wise accuracy was 83.8% for miT (95%CI: 0.70–0.95), 100% for miN, and 89.1% for miM (95%CI: 0.77–0.97).

ChatGPT-4o *underestimated* one miT category, and *overestimated* 5 miT and 4 miM categories. All T and M stages misclassifications resulted from information that was not explicitly stated or insufficiently captured in the reports. For miT, a frequent cause of overestimation was that ChatGPT-4o inferred capsular infiltration and classified cases as T3, even though capsular breach was not described (*n* = 4), detailed information of this shift can be seen in Table 3. For further details on misclassification of PET/MRI reports, see Supplementary Table S1. Additionally, some cases were underestimated because subtle distinctions between unifocal (u) or multifocal (m) tracer uptake were not captured (*n* = 2). For miT, the interreader agreement between the two readers from PET/MRI reports was κ = 0.59 (CI: 0.41–0.75). The agreement was κ = 0.58 (CI: 0.30–0.81) for miN and κ = 0.65 (CI: 0.35–0.85) for miM. Agreement between ChatGPT-4o and the validation reading was κ = 0.78 (CI: 0.62–0.92) for miT, κ = 1.00 for miN, and κ = 0.78 (CI: 0.55–0.95) for miM.

**Fig. 3:**
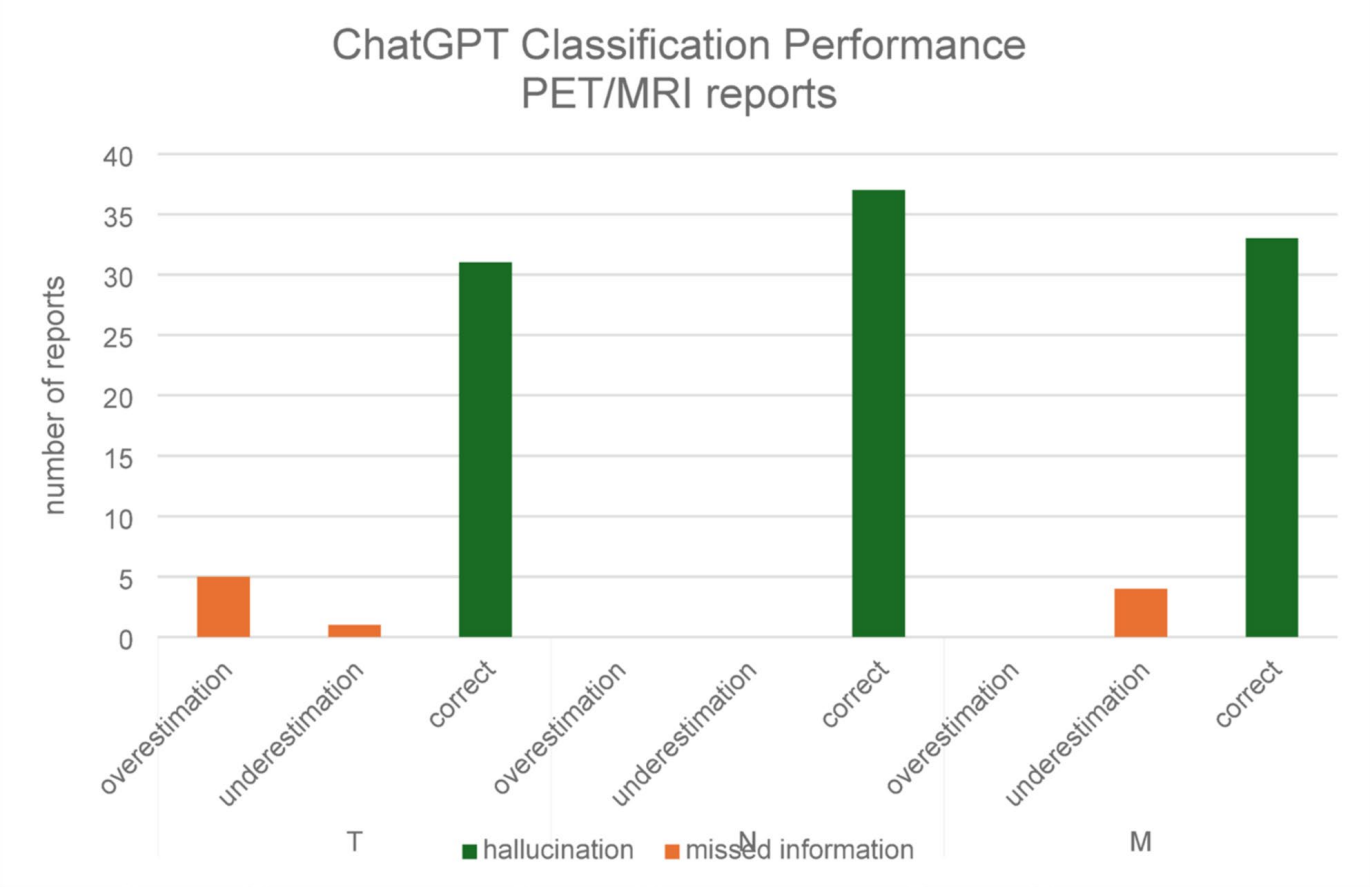
Performance of ChatGPT-4o for molecular imaging (mi) tumour (T), node (N) and metastases (M) classification applied on PSMA PET/MRI reports

For miN, underestimation occurred when ChatGPT-4o did not fully interpret ambiguous formulations such as “pathologic lymph nodes in the small pelvis extending up to the paracaval region.” (Fig. 3).

**Table 3:** ChatGPT-4o vs. human validation reading for molecular imaging (mi) tumour (T) classification applied on PSMA PET/MRI reports

PSMA PET/MRI			Incorrect predictions											
Validation	Total count	Correct prediction	Overestimations						Underestimations					
			T2u	T2m	T3a	T3b	T3c	T4	T0	T2u	T2m	T3a	T3b	T3c
T0	15	**15**	0	0	0	0	0	0	0	0	0	0	0	0
Tr	9	**8**	0	0	0	0	0	**1**	0	0	0	0	0	0
T2u	5	**2**	**1**	0	**2**	0	0	0	0	0	0	0	0	0
T2m	4	**2**	0	0	**1**	0	0	0	0	**1**	0	0	0	0
T3a	0	na	na	na	na	na	na	na	na	na	na	na	na	na
T3b	1	**1**	0	0	0	0	0	0	0	0	0	0	0	0
T4	3	**3**	0	0	0	0	0	**0**	0	0	0	0	0	0

Among the 17 PET/MRI images eligible for PRIMARY scoring, ChatGPT-4o achieved an accuracy of 88.1% (95%CI: 0.69-1.00) with an interreader agreement of κ = 0.96 (CI: 0.78-1.00). when comparing the validation reading with ChatGPT-4o.

### Comparison of miTNM and PROMISE results for PET/CT and PET/MRI

ChatGPT-4o achieved significantly higher accuracy for PET/MRI compared to PET/CT in T-stage classification (83.8% vs. 57.7%; OR = 3.83, 95% CI: 1.34–12.69, *p* = 0.006) and N-stage classification (100% vs. 85.9%, *p* = 0.014). In contrast, performance was comparable for M-stage classification (89.1% vs. 95.7%; OR = 0.84, 95% CI: 0.20–4.19, *p* = 0.748) and PRIMARY score assessment (70.4% vs. 88.1%; OR = 0.43, 95% CI: 0.05–2.14, *p* = 0.315). Overall, ChatGPT-4o demonstrated high accuracy for miTNM classification, with significantly more accurate performance on PET/MRI reports compared to PET/CT reports (91.0% vs. 79.8%; OR: 2.80 95% CI: 1.32–6.51, *p* = 0.003).

### Accuracy analysis by clinical indications (initial staging vs. recurrence assessment) for PET

**Fig. 4:**
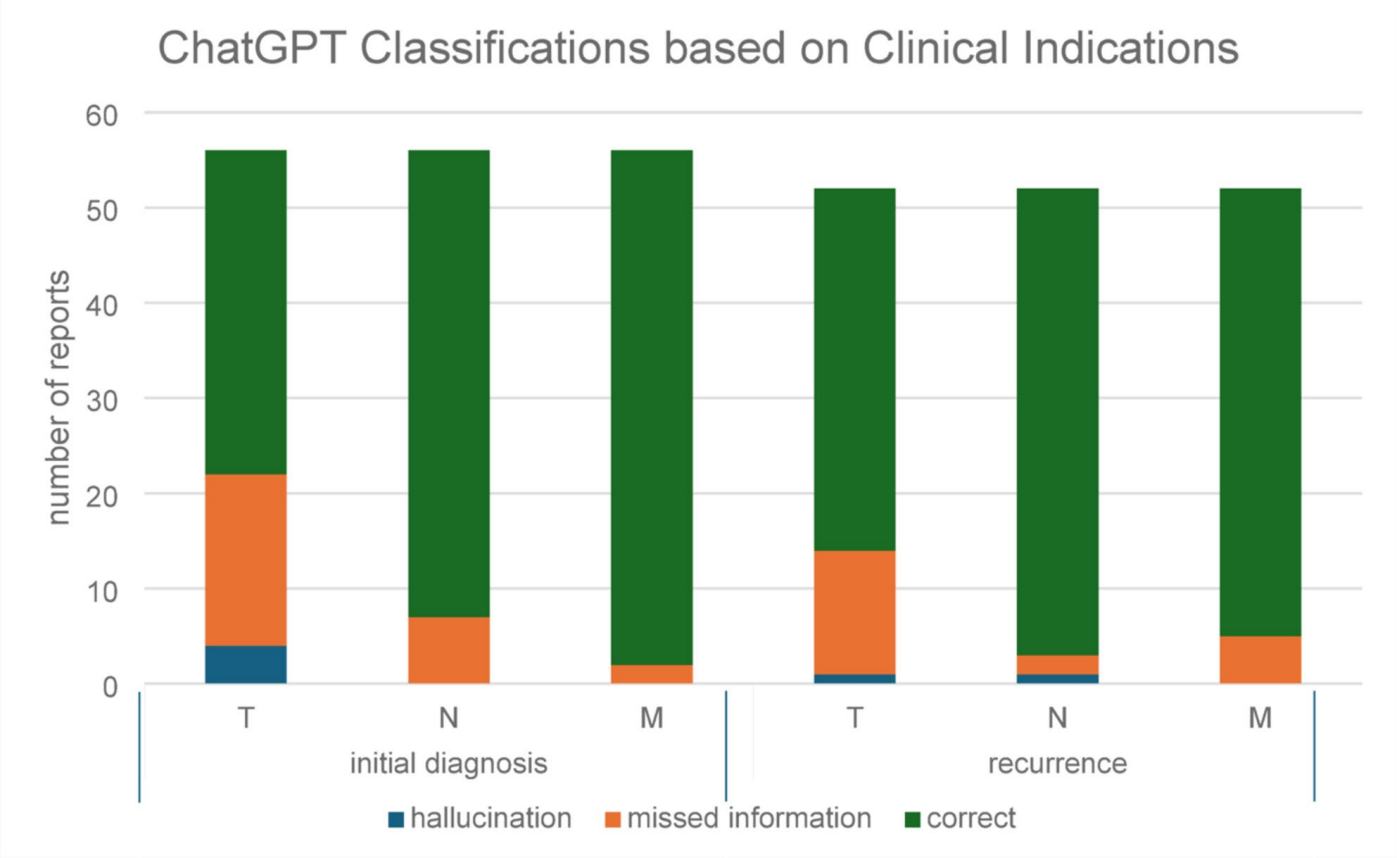
Performance of ChatGPT-4o for molecular imaging (mi) tumour (T), node (N) and metastases (M) classification among clinical indications recurrence vs. initial staging

For initial staging (*n* = 56), overall miTNM classification accuracy was 85.9% (95% CI: 0.74–0.86). Component-wise accuracy was 60.7% for miT, 87.5% for miN, and 96.4% for miM. For recurrence (*n* = 52), overall miTNM classification accuracy was 81.5% (95% CI: 0.79–0.90). Component-wise accuracy was 73.0% for miT, 94.2% for miN, and 90.4% for miM. Comparisons between initial staging and recurrence showed no significant differences for miT classification (OR = 1.76, 95% CI: 0.74–4.27, *p* = 0.228), miN classification (OR = 2.03, 95% CI: 0.50–9.83, *p* = 0.362), or miM classification (OR = 0.45, 95% CI: 0.07–2.23, *p* = 0.312). Overall, ChatGPT-4o performance was comparable between both clinical indications (OR = 1.31, 95% CI: 0.73–2.52, *p* = 0.315), as illustrated in Fig. 4. Hallucinations were observed in only six cases.

### Plausibility assessment of ChatGPT-4o explanations to results

The factual plausibility of ChatGPT-4o’s explanations were rated consistently high among modalities for miTNM and PRIMARY scoring. Mean Likert scores were:

- miT = 4.8 ± 0.37, miN = 4.9 ± 0.30, miM = 4.8 ± 0.30 for PET/CT.
- miT = 4.9 ± 0.38, miN = 4.9 ± 0.38, miM = 4.9 ± 0.51 for PET/MRI.
- 4.1 ± 1.4 for PRIMARY in PET/CT.
- 4.9 ± 0.32 for PRIMARY in PET/MRI.

## Discussion

To our knowledge, this is the first study to evaluate the utility of LLMs for the automated extraction of PROMISE V2-based classifications from unstructured PSMA PET/CT and PET/MRI reports in patients with prostate cancer. Our results demonstrate that ChatGPT-4o enables extraction of miTNM and PRIMARY classifications from free-text reports in clinical routine, with high accuracy for nodal (miN) and distant metastasis (miM) staging across both modalities. However, accuracy for miT classification was significantly higher for PSMA-PET/MRI reports.

For PSMA-PET/CT, the accuracy was moderate for miT (57.7%), but high for miN (85.9%) and miM (95.7%). For PSMA-PET/MRI, the accuracy was sufficient for miT (83.8%), miN (100%) and miM (89.1%). These results are consistent with Yuan et al., who reported high accuracy rates of up to 94% for TNM staging using LLMs in prostate cancer [17]. However, classification of the primary tumour (miT) was less accurate in our PET/CT cohort. One explanation is the frequent use of vague or missing information on extracapsular extension/no extracapsular extension (T3) and potential infiltration (T4), as well as missing or vague description on unifocal or multifocal presence of cancer lesions in free-text reports, making precise staging difficult. For instance, ChatGPT-4o often underrated T stage due to missing detailed information on uni-/multifocality of the primary tumor, which has been similarly observed by Nakamura et al. who reported high accuracy for N (78.9%) and M (86.7%) staging but markedly lower accuracy for T staging (52.2%) in lung cancer [18]. Suzuki et al., identified a related issue in pancreatic cancer reports, where underestimation of T4 tumours occurred when vessel “contact” was described without explicitly stating vascular invasion [19].

These findings suggest that the performance of LLMs is closely linked to the precision and clarity of the underlying report, highlighting a significant bottleneck for the implementation of automated structured reporting and underscoring the importance of standardized phrasing within free-text reports.

Therefore, we emphasize the importance of either using highly structured language within free-text reports or explicitly incorporating linguistic nuances, tailored to each center’s typical terminology into the prompt to minimize misclassification. Even the incorporation of domain specific knowledge into the prompt already demonstrated high performance through expert-informed prompt engineering, achieving a T-staging accuracy of 91.3% [20].

In our analysis ChatGPT-4o achieved notably higher miT and miN accuracy from PET/MRI reports compared to PET/CT reports (miT: 83.8% vs. 57.7%, *p* = 0.006; miN: 100% vs. 85.9%, *p* = 0.014), interestingly, ChatGPT-4o showed higher interreader agreement compared to the validation set than interreader agreement between the two human readers for PET/MRI reports (κ = 0.78 vs. κ = 0.59) and lower agreement for PET/CT reports (vs. 0.38 vs. κ = 0.42) for T stage and also for N stage (PET/MRI: κ = 1.00 vs. κ = 0.58; PET/CT: κ = 0.69 vs. κ = 0.80). miM accuracy remained comparable for PET/CT and PET/MRI (89.1% vs. 95.7%, *p* = 0.748) and ChatGPT-4o showed higher agreements compared to the validation sets than the agreements between the two human readers in both modalities (PET/MRI: κ = 0.78 vs. κ = 0.65; PET/CT: κ = 0.90 vs. κ = 0.79). This variability in performance has also been reported by Alalawi et al., who demonstrated that GPT-4o’s performance varies considerably across different radiology subspecialties and imaging modalities [21]. However, the observed differences in miTNM classification performance between PET/MRI and PET/CT in our study should be interpreted with caution, given the limited number of PET/MRI reports included.

From a clinical perspective, accurate miT classification is critical, as misclassification can lead to inappropriate treatment strategies. In our study, most errors, which were mainly minor and caused by missing detailed information of tumour extent for accurate use of PROMISE in free-text-reports, were underestimations. These observations underline the importance of not relying solely on automated extraction tools yet, making human oversight remain essential to ensure clinical accuracy and patient safety.

Given the limited diagnostic value of PSMA-PET/CT for primary prostate tumours [22–24], the PRIMARY score was developed to enhance local staging accuracy and guide PSMA-PET-guided biopsy, particularly in newly diagnosed or biopsy-naïve patients [25]. Applying the PRIMARY score to both modalities, our results demonstrated comparable accuracy for PET/MRI reports (88.1%) compared to PET/CT (70.4%). The slightly superior performance of PET/MRI (even though there was no significant difference) in the PRIMARY score may also be attributed to its higher soft-tissue contrast, which allows for more precise description of the anatomical localization and consequently reporting of the primary tumour extent e.g. exact discrimination of peripheral vs. transitional zones).

Across all parameters, the rationale for each classification using a five-point Likert scale was rated very high between 4.1 and 4.9. This supports its use as an explainable AI system in clinical practice. However, rather than replacing physicians, ChatGPT-4o can function as an augmented intelligence assistant, helping structure complex reports, support tumour board preparation, and reduce time-to-decision in high-throughput settings – especially as PSMA-PET/CT is gaining widespread clinical use following the EMA/FDA approval of [^177^Lu]Lu-PSMA-617 (Pluvicto) and growing demand for PSMA-targeted diagnostics and therapy. Moreover, ChatGPT-4o could support not only structured data extraction for clinical use, but also for clinical trials to ensure adherence, particularly if standardized reporting schemes incorporating PSMA response metrics such as PSMA-PET Progression (PPP) and the Response Evaluation Criteria in PSMA-PET/CT (RECIP) are used. For example, Andrei et al. have shown that the RECIP criteria are highly relevant for assessing overall survival (OS), as patients classified as having RECIP-progressive disease exhibit significantly lower OS compared to those with stable disease or a therapy response [26, 27]. Prior studies, such as those by Fink et al. and Bhayana et al., have already highlighted this role in radiology [5, 14].

Several limitations must be acknowledged. First, this was a retrospective single-center study using German-language reports, which may limit generalizability. Although ChatGPT-4o is multilingual, the combination of German-language source reports and English-language prompts could have introduced subtle comprehension challenges [28]. Even though we pre-evaluated reports using the prompt in both German and English and no differences in the output were observed between the two languages, larger cohorts are needed to identify multilingual differences. Second, both [^68^Ga]- and [^18^F]-labelled PSMA tracers were included, which may differ in biodistribution and influence interpretation, especially for the PRIMARY score, as the score 5 was originally developed and validated for ^68^[Ga]-PSMA-11 PET/CT imaging. Third, sample size was significantly smaller for PET/MRI, mainly because numbers of PET/MRIs in our centre are generally low compared to PET/CTs, which may influence statistical power. Moreover, we used the web interface and not the API, which might lead to slightly variable results as the parameters such as temperature or seed are not fixed in the web application. Ultimately, LLMs are improving fast with newer versions being introduced regularly. Therefore, ChatGPT-4o might already be outdated at time of publication.

## Conclusion

Overall, this study supports the feasibility of using ChatGPT-4o as a clinical companion for structured extraction of PROMISE V2-based classifications from PSMA PET reports. By extracting miN and miM as well as the PRIMARY scores from unstructured clinical free-text reports, ChatGPT-4o offers a scalable solution to improve standardization and efficiency in oncologic imaging workflows. Caution is required for T-stage classification, as our results show limited performance for extraction of miT, mainly due to misinterpretation of ambiguous language in free-text reports (for instance, the use of the plural term “uptakes” in the projection of the prostate led ChatGPT-4o to assign T2u, while readers classified the finding as T2m). This underscores that insufficiently structured or imprecise reporting can significantly impair reliable information extraction by LLMs; they cannot compensate for vague or ambiguous input. Accurate performance depends on clear and explicit textual information. However, given the increasing clinical relevance of PSMA PET/CT – especially in the context of PSMA-targeted therapies – such approaches may offer added value in daily practice, particularly under conditions of high workload or limited specialist availability. Further prospective and multi-center validation studies will be essential to confirm generalizability and ensure safe implementation, particularly in high-stakes settings such as oncological stagings. Nonetheless, this work provides a first step toward leveraging language-based AI to support structured, explainable, and efficient reporting in molecular imaging.

## Supplementary Information

Supplementary Material 1

## Data Availability

The datasets generated during the current study are available from the corresponding author on reasonable request.

## Abbreviations

AI: Artificial Intelligence
CI: Confidence Interval
CT: Computed Tomography
DWI: Diffusion Weighted Imaging
EMA: European Medicines Agency
FDA: Food and Drug Administration
GPT: Generative Pre–Trained Transformer
HASTE: Half–Fourier Acquisition Single–shot Turbo spin Echo
LLM: Large Language Model
mi: Molecular Imaging
miM: Molecular Imaging Metastasis Classification
miN: Molecular Imaging Node Classification
miT: Molecular Imaging Tumour Classification
miTNM: Molecular Imaging Tumour–Node–Metastasis Classification
MRI: Magnetic Resonance Imaging
OS: Overall Survival
PET: Positron Emission Tomography
PPP: PSMA–PET Progression
PRIMARY: Prostate Imaging Reporting system for Intra–prostatic PSMA uptake
PROMISE: Prostate Cancer Molecular Imaging Standardized Evaluation
PSA: Prostate Specific Antigen
PSMA: Prostate–Specific Membrane Antigen
RECIP: Response Evaluation Criteria in PSMA–PET/CT
ROI: Region of Interest
SD: Standard Deviation
SUV / SUVmax: Standardized Uptake Value / maximum Standardized Uptake Value
T1: VIBE–T1–weighted Volumetric Interpolated Breath–hold Examination
TNM: Tumour–Node–Metastasis
u / m: Unifocal / Multifocal
