# Supplementary material for: Can chatGPT-4o reliably standardize PSMA PET/CT and PET/MRI reports using PROMISE V2 criteria? - An exploratory study
Source: EJNMMI Res. 2026 Jun 26;16:101. doi: 10.1186/s13550-026-01475-z (PMC13309584; doi:10.1186/s13550-026-01475-z)
Supplement: Supplementary file 1 — Supplementary Material 1 [file 13550_2026_1475_MOESM1_ESM.docx]

| **Modality** | **Chat-GPT Classification** | **Reading Classification** | **Description of the primary tumor in report** | **Chat-GPTs given rational** |
| --- | --- | --- | --- | --- |
| MRI | T4 | Tr | Circumscribed local recurrence in the former prostatic bed following prostatectomy. | There is a local recurrence with contact to both the bladder base and the anterior rectal wall, which indicates invasion of adjacent organs. According to PROMISE V2, this corresponds to miT4. |
| MRI | T2u | T2m | Tumour-suspicious focal tracer uptakes in the prostate, up to 6 mm in size (basal ventral, immediately inferior to the bladder base). | There is a focal PSMA-avid lesion in the prostate, basal midline near the bladder base, measuring approximately 6 mm, without evidence of extraprostatic extension. This corresponds to unifocal, organ-confined tumour, which is classified as miT2u. |
| MRI | T3a | T2u | Consistent with the previous MRI, highly suspicious focal tracer uptake in the prostate apically on the left with SUVmax of approximately 16.3 (peripheral zone mid and peripheral zone posterolateral). Broad-based contact with the capsule and the immediately adjacent seminal vesicles, the latter without definite evidence of infiltration. | The report describes a highly suspicious uptake in the left apical peripheral zone with broad contact to the prostate capsule and adjacent seminal vesicles, but no definitive infiltration of the seminal vesicles. According to PROMISE V2, broad capsular contact without clear extracapsular extension into adjacent organs or seminal vesicles corresponds to miT3a. |
| MRI | T3a | T2m | Intraprostatic uptake apically on the left with SUVmax 10 (peripheral zone mid and transition zone apical left), as well as centrally on the left with SUVmax 8.7 (peripheral zone mid and transition zone mid left) and 9.3 (anterior peripheral zone left), the latter with a high suspicion of capsular infiltration. Overall, however, no definite evidence of extracapsular extension. | There is a suspicion of capsular infiltration in the left peripheral anterior zone, which aligns with miT3a in PROMISE V2. Although no definitive extracapsular extension is confirmed, a "suspicion of capsule infiltration" meets criteria for miT3a. |
| MRI | T3a | T2u | Intense comma-shaped tracer uptake in the prostate apically on the right ventrolaterally (PZa and anterior stroma) with SUVmax up to 21.7, with capsular infiltration, but without definite evidence of extracapsular extension. | The report describes a highly PSMA-avid lesion in the apical right ventrolateral prostate with capsular infiltration, but no evidence of extracapsular extension. According to PROMISE V2, miT3a includes tumours with capsular infiltration but without invasion into the seminal vesicles (miT3b) or adjacent structures (miT4). |
| MRI | T2m | T2u | PSMA uptake in projection of the prostate gland in the presence of histologically confirmed prostate carcinoma, with SUVmax up to 5.0. | PSMA uptake in the prostate itself (SUVmax 5.0) without mention of extraprostatic extension, seminal vesicle infiltration, or bladder/rectum invasion. |
| CT | T2u | Tr | Suspicious PSMA uptake in projection of a soft tissue mass in the prostatic bed consistent with a local recurrence. | The report describes a PSMA uptake in a soft tissue mass in the prostatic bed consistent with a local recurrence (post-prostatectomy recurrence). |
| CT | T2u | T2m | Areas of increased PSMA expression in projection of the prostate gland, with SUVmax up to 5.5. | There is PSMA uptake in the prostate itself (SUVmax 5.5), with no mention of extracapsular extension, seminal vesicle involvement, or invasion into adjacent structures. Therefore, classified as localized intraprostatic tumour (miT2u = unilateral intraprostatic uptake without evidence of extraprostatic extension; assuming unilateral as no bilateral involvement is specified). |
| CT | T2u | Tr | Soft-tissue prominence in the true pelvis caudal to the bladder (approximately 1.0 × 1.4 cm axial, SP -1081), SUVmax 7.6 | PSMA uptake in a soft tissue mass caudal to the bladder is consistent with a local recurrence (post-prostatectomy recurrence). |
| CT | T2m | T2u | In projection of the prostate gland in the presence of histologically confirmed prostate carcinoma, with SUVmax up to 3.7. | PSMA uptake in the prostate itself (SUVmax 3.7) without mention of extraprostatic extension, seminal vesicle infiltration, or bladder/rectum invasion. |
| CT | T2u | T2m | Suspicious increased tracer uptakes in projection of the hypertrophic prostate in the presence of histologically confirmed prostate carcinoma. | The report describes an enlarged prostate with suspected PSMA uptake confined to the prostate. There is no mention of extracapsular extension, seminal vesicle invasion, or other organ involvement. This supports organ-confined, unilateral disease, consistent with miT2u. |
| CT | T2u | T2m | Suspicious increased tracer uptakes in projection of the hypertrophic prostate in the presence of histologically confirmed prostate carcinoma | There is focal PSMA uptake limited to the prostate without evidence of extracapsular extension, seminal vesicle invasion, or involvement of adjacent organs. The uptake is described as localized (“in projection onto the prostate”), and no other local extension is mentioned. Thus, the finding corresponds to organ-confined, unilateral disease, consistent with miT2u. |
| CT | T2u | T2m | Suspicious increased tracer uptakes in projection of the hypertrophic prostate in the presence of histologically confirmed prostate carcinoma wit SUVmax up to 23.3. | There is tumour-suspect PSMA uptake confined to the prostate with no mention of extracapsular extension, seminal vesicle invasion, or adjacent organ involvement. The prostate is described as “hypertrophied” with PSMA uptake (SUVmax 23.3), but the lesion appears organ-confined and unilateral. Thus, the finding is consistent with miT2u. |
| CT | T2u | T2m | In projection of the prostate gland in the presence of histologically confirmed prostate carcinoma, with SUVmax up to 5.5. | The report describes increased PSMA uptake confined to the prostate without signs of extraprostatic extension or involvement of adjacent organs. This fits miT2u, indicating unifocal or unclear multifocal intraprostatic tumour activity. |
| CT | T2u | T2m | Prostatic hyperplasia with heterogeneous mildly to moderately increased PSMA expressions. | PSMA uptake is localized to the right peripheral zone of the prostate without evidence of extracapsular extension or involvement of other zones. This indicates a unifocal intraprostatic lesion. |
| CT | T3b | T2m | Clinical information: Diffuse and highly carcinoma-suspicious signal alterations involving nearly the entire prostate, with relative sparing of the right peripheral zone and strong suspicion of infiltration into the seminal vesicle insertions. Infiltration was not seen on PT/CT. Overall enlarged prostate with increased diffuse tracer uptake, SUVmax 16.9. | The PSMA-PET/CT shows diffuse tracer uptake throughout the prostate with MRI evidence of infiltration into the seminal vesicle insertions (PIRADS V), indicating seminal vesicle involvement consistent with miT3b. |
| CT | T2u | T2m | Overall enlarged prostate with increased tracer uptake in the right peripheral region, SUVmax 24.9. | There is focal, intense PSMA uptake in the right peripheral zone of the prostate (SUVmax 24.9), without imaging signs of extracapsular extension or seminal vesicle infiltration. This corresponds to multifocal intraprostatic disease, consistent with miT2m. |
| CT | T2u | T2m | Overall enlarged prostate with increased tracer uptakes, up to SUVmax 7.8. | There is diffuse PSMA uptake in an overall enlarged prostate (SUVmax 7.8), but no evidence of extracapsular extension, seminal vesicle invasion, or bladder/rectal infiltration is described. The uptake appears unifocal or diffuse without signs of higher-stage local invasion, supporting miT2u. |
| CT | T2u | T2m | Diffuse increased tracer uptakes in projection of the prostate in the presence of histologically confirmed prostate carcinoma, up to SUVmax 5.2. | PSMA uptake is confined to the prostate without evidence of extracapsular extension or seminal vesicle/bladder invasion. There is no mention of invasion into adjacent organs or structures. Thus, the tumour is localized within the prostate (miT2), unspecified in terms of lobes (u = unspecified). |
| CT | T2u | T2m | Overall enlarged prostate with increased tracer uptakes, up to SUVmax 4.5 | Increased PSMA uptake confined to the prostate without signs of extraprostatic extension (e.g., no seminal vesicle, bladder, or adjacent organ invasion described), fitting with miT2u (tumour confined within the prostate, unspecified lobes). |
| CT | T3a | T2m | Areas in projection of the enlarged prostate in the presence of histologically confirmed prostate carcinoma, with uptakes up to SUVmax 5.1. | The report mentions organ-transcending (extraprostatic) tumor growth, consistent with miT3a (extracapsular extension without seminal vesicle invasion). |
| CT | T3a | T2m | Areas in projection of the right lobe of the prostate in the presence of histologically confirmed prostate carcinoma, wih SUVmax up to 21.5. | The clinical information states "lokal fortgeschrittenes Karzinom, T3", indicating extracapsular extension without evidence of invasion into other structures (such as seminal vesicles or adjacent organs). Since no seminal vesicle (miT3b), bladder/rectum invasion (miT4), or local recurrence (miTr) is described, the highest appropriate T stage is miT3a. |
| CT | T2u | T2m | Diffuse increased tracer uptakes in projection of the enlarged prostate, up to SUVmax 6.2. | The PSMA uptake is described as diffuse without mention of extracapsular extension, seminal vesicle involvement, or invasion into adjacent organs. This suggests organ-confined disease, fulfilling criteria for miT2. Since the report describes "diffuse" uptake (not focal), it is categorized as miT2u (unifocal or multifocal unclear), as no distinct multifocal lesions were described. |
| CT | T2u | T2m | Diffuse increased tracer uptakes in projection of the enlarged prostate. | PSMA uptake is described projection of the enlarged prostate with no mention of extracapsular extension, seminal vesicle invasion, or adjacent organ involvement, indicating organ-confined disease (miT2). The description does not specify clear multifocality, so it is classified as miT2u. |
| CT | T2m | T2u | Overall enlarged prostate with increased tracer uptake in the right caudal region, SUVmax 12.5. | The PSMA PET/CT shows increased uptake in an enlarged prostate, particularly in the right caudal region, with a SUVmax of 12.5. No evidence is provided for extracapsular extension, seminal vesicle invasion, or invasion of adjacent structures. The wording suggests multifocal intraprostatic involvement → miT2m. |
| CT | T2u | T2m | Suspicious increased tracer uptakes in projection of the enlarged prostate up to SUVmax 44,8, in the presence of histologically confirmed prostate carcinoma. | The report describes a generally enlarged prostate with strong PSMA uptake, but no mention of extracapsular extension or seminal vesicle invasion. This corresponds to miT2u. |
| CT | T2u | T2m | Suspicious increased tracer uptakes in projection of the enlarged prostate with SUVmax up to 11,4. | The report describes an enlarged prostate with PSMA uptake and no evidence of extracapsular extension or seminal vesicle invasion. This corresponds to miT2u. |
| CT | T2u | T2m | Suspicious increased tracer uptakes in projection of the enlarged prostate up to SUVmax 12.2. | The report describes a pathologically enlarged prostate with PSMA uptake, with no mention of extracapsular extension or seminal vesicle involvement. This corresponds to miT2u. |
| CT | T2u | T2m | Suspicious increased tracer uptakes in projection of the enlarged prostate up to SUVmax 21.2. | The report describes PSMA uptake confined to an enlarged prostate without any mention of extracapsular extension or seminal vesicle invasion. This corresponds to miT2u. |
| CT | T2u | T2m | Suspicious increased tracer uptakes in projection of the enlarged prostate up to SUVmax 18.1. | The report describes PSMA uptake confined to the prostate with no imaging evidence of extracapsular extension or seminal vesicle invasion. While there was a clinical suspicion of extracapsular extension, the PET/CT findings do not confirm this. According to PROMISE V2, classification is based on imaging findings, so this corresponds to miT2u. |
| CT | T2u | T2m | Suspicious increased tracer uptakes in projection of the hypertrophic prostate with point of maximum uptake in the left prostatic lobe, SUVmax 40.5. | The report describes PSMA uptake confined to the prostate without imaging evidence of extracapsular extension or seminal vesicle invasion. This corresponds to miT2u. |
| CT | T2u | T2m | Suspicious increased tracer uptakes in projection of the prostate with SUVmax up to 20.4. | The report describes PSMA uptake confined to the prostate with no imaging evidence of extracapsular extension or seminal vesicle invasion. This corresponds to miT2u |
| CT | T2u | T2m | Suspicious increased tracer uptakes in projection of the enlarged prostate with SUVmax up to 6.3. | The report describes PSMA uptake confined to an enlarged prostate with no imaging evidence of extracapsular extension or seminal vesicle invasion. Although the clinical TNM staging mentioned cT2c, the PROMISE classification is based on the imaging findings, which here correspond to miT2u. |
| CT | T2u | T2m | Suspicious increased tracer uptakes in projection of the enlarged prostate with SUVmax up to 5.7. | The report describes PSMA uptake confined to an enlarged prostate without imaging evidence of extracapsular extension or seminal vesicle invasion. This corresponds to miT2u. |
| CT | T3b | T2m | Clinical information: strong suspicion of infiltration into the right neurovascular bundle and right seminal vesicle, which was not seen on PT/CT. Suspicious increased tracer uptakes in projection of the enalrged prostate with SUVmax up to 12.0. | The clinical report and indication mention suspected infiltration of the right neurovascular bundle and right seminal vesicle, and the PET finding supports high PSMA uptake in the prostate (SUVmax 12.0). Even though the PSMA report itself does not directly state seminal vesicle invasion, the combination of clinical suspicion and PSMA uptake localized in a high-risk context is best classified conservatively as miT3b (seminal vesicle involvement). |
| CT | T3b | T2m | Clinical information: strong suspicion of infiltration into the seminal vesicle, which was not seen on PT/CT. Suspicious increased tracer uptakes in projection of the prostate with SUVmax up to 6.0. | The clinical staging and report indicate suspected infiltration of the seminal vesicle (cT3b), which is consistent with miT3b. The PSMA-PET supports the presence of intraprostatic disease but doesn't contradict the cT3b assumption. |

*Table S1. Case based analysis of misclassified T categories.*
